# ﻿Illustrated keys and a DNA barcode reference library of the amphibians and terrestrial reptiles (Amphibia, Reptilia) of São Tomé and Príncipe (Gulf of Guinea, West Africa)

**DOI:** 10.3897/zookeys.1168.101334

**Published:** 2023-06-27

**Authors:** Luis Miguel Pires Ceríaco, Mariana Pimentel Marques, Ana Carolina Andrade de Sousa, Joana Veríssimo, Pedro Beja, Sónia Ferreira

**Affiliations:** 1 Departamento de Zoologia e Antropologia (Museu Bocage), Museu Nacional de História Natural e da Ciência & Instituto de Investigação Científica Tropical (IICT), Universidade de Lisboa, Rua da Escola Politécnica, 58, 1269-102 Lisboa, Portugal; 2 Universidade Federal do Rio de Janeiro, Museu Nacional, Departamento de Vertebrados, Av. Bartolomeu de Gusmão 875, São Cristóvão, 20941-160 Rio de Janeiro, Brasil; 3 CIBIO, Centro de Investigação em Biodiversidade e Recursos Genéticos, InBIO Laboratório Associado, Campus de Vairão, Universidade do Porto, 4485-661 Vairão, Portugal; 4 BIOPOLIS Program in Genomics, Biodiversity and Land Planning, CIBIO, Campus de Vairão, 4485-661, Vairão, Portugal; 5 Departamento de Biologia, Faculdade de Ciências da Universidade do Porto, Rua do Campo Alegre 1021, 4169-007 Porto, Portugal; 6 Grutas da Moeda e Fátima Limitada, Largo das Grutas da Moeda, sem número, 2495-028 São Mamede, Portugal; 7 CIBIO, Centro de Investigação em Biodiversidade e Recursos Genéticos, InBIO Laboratório Associado, Instituto Superior de Agronomia, Universidade de Lisboa, Lisboa, Portugal

**Keywords:** Biological surveys, conservation, Cytochrome c oxidase subunit I (COI), DNA metabarcoding, environmental DNA, Herpetofauna, Oceanic Islands

## Abstract

The herpetofauna of São Tomé and Príncipe consists of nine species of amphibians, all endemic, and 21 species of terrestrial reptiles, of which 17 are endemic. Our current knowledge regarding its natural history, ecology, and distribution is limited. Here two important tools are provided to support researchers, conservationists, and local authorities in the identification of the country’s herpetofauna: an illustrated key to the herpetofauna of the two islands and surroundings islets and a DNA barcode reference library. The keys allow a rapid and unambiguous morphological identification of all occurring species. The DNA barcodes for the entire herpetofauna of the country were produced from 79 specimens, all of which are deposited in museum collections. The barcodes generated are available in online repositories and can be used to provide unambiguous molecular identification of most of the species. Future applications and use of these tools are briefly discussed.

## ﻿Introduction

The herpetofauna of São Tomé and Príncipe, a small insular country in the Gulf of Guinea, West Africa, has been the subject of an intense taxonomic and systematic revision in the last decade (see Bell et al. 2022a and Ceríaco et al. 2022 for an overview). Hence, the country’s herpetofauna is currently one of the best known in Africa, with nine recorded species of amphibians (Bell et al. 2022a) and 21 recorded species of reptiles (Ceríaco et al. 2022). Of these, all of the amphibian species are endemic to their respective island (Bell et al. 2022a), while 17 out of 21 reptiles are also endemic (Ceríaco et al. 2022).

Recent research efforts have focused on the taxonomic revision, systematic placements, and biogeographic patterns associated with the amphibians and terrestrial reptiles of the country. However, not much attention has been given to their natural history, distribution, and ecological relationships. Besides some anecdotal data available in taxonomic papers, only a few studies provided details on the trophic ecology of São Tomé and Príncipe herpetofauna (Manaças 1958, 1973; Delêtre and Measey 2004; Jones et al. 2006; Cascio 2022; Sousa et al. 2022). Recent research has focused on the trophic ecology of the Tinhosa Grande islet *Trachylepisadamastor* population (Sousa et al. 2022), and of the Príncipe Island endemic *Feyliniapolylepis* (Cascio 2022). No data is currently available on predation by either native or introduced predators on the amphibians and reptiles of São Tomé and Príncipe. Contrary to birds (Melo et al. 2022), plants (Dauby et al. 2022), and sea turtles (Ferreira-Airaud et al. 2022), data on the habitat association of São Tomé’s amphibians and reptiles is scarce and limited to *Hyperolius* tree frogs (Strauss et al. 2018). These data gaps preclude a more complete understanding of the ecology of these islands’ ecosystems, its contextualization into broader scenarios, as well as the implementation of data-driven conservation strategies (Bell et al. 2022b; Soares et al. 2022).

Overcoming these knowledge gaps demands additional natural history and ecological studies using a plethora of field techniques, from traditional field surveys to the use of modern techniques such as DNA barcoding and metabarcoding. To contribute to a more accurate and easy identification of the amphibians and terrestrial reptiles of São Tomé and Príncipe, here we provide an illustrated identification key as well as a DNA barcode reference library.

## ﻿Materials and methods

### ﻿Field sampling and natural history collections

Specimens were collected in São Tomé and Príncipe islands and surrounding islets (Tinhosa Grande) following the traditional techniques used for herpetological surveys (see Simmons 2015) and in accordance with local and international legislation (see permits information in the acknowledgments). The collected specimens were fixed in the field with 10% buffered formalin and transferred to 70% ethanol for long-term preservation. Liver tissue was removed before formalin fixation and preserved in 95% ethanol for storage. Collected specimens were deposited in the Museu Nacional de História Natural e da Ciência (**MUHNAC**; Lisbon, Portugal) amphibians (MUHNAC/MB04) and reptiles (MUHNAC/MB03) collections, as well as in the Museu de História Natural e da Ciência da Universidade do Porto (**MHNCUP**; Porto, Portugal) amphibians (MHNCUP/AMP) and reptiles (MHNCUP/REP) collections (Table 1). Additional specimens housed in the collections of the Instituto de Investigação Científica Tropical (**IICT**; Lisbon, Portugal, see Ceríaco et al. 2021a) and the California Academy of Sciences (**CAS**; San Francisco, USA) were also consulted and sequenced (Table 1).

**Table 1.** List of taxa and respective specimens that were collected and DNA barcoded (Cytochrome c oxidase subunit I, 658 bp) . *Indicate species with previously available BINs. See Materials and methods section for collection abbreviations.

Species	Specimen ID		Locality [coordinates, elevation]	BOLD BIN	GenBank accession number
	Museum number	BOLD code			
**AMPHIBIA**					
**ORDER ANURA**					
**Family Arthroleptidae**					
**Genus *Leptopelis***					
*Leptopelispalmatus* (Peters, 1868)*	MB04-000792	IAHTP015-22	Príncipe Island: Campo Político [1.6448, 7.3990, 202 m]	BOLD:ADB9336	OQ174598
	MB04-000791	IAHTP014-22	Príncipe Island: Pico Mesa, base [1.5876, 7.3571, 316 m]		OQ174595
	MB04-000788	IAHTP013-22	Príncipe Island: Campo Político [1.6448, 7.3990, 202 m]		OQ174628
	MHNC-UP-AMP 401	IAHTP068-22	Príncipe Island: Biosphere Reserve, trail to Santa Joaquina overview [1.6048, 7.4018, 315 m]		OQ174604
**Family Hyperoliidae**					
**Genus *Hyperolius***					
*Hyperoliusdrewesi* Bell, 2016*	MHNC-UP-AMP 397	IAHTP028-22	Príncipe Island: Biosphere Reserve, trail to Santa Joaquina overview [1.6048, 7.4018, 315 m]	BOLD:ADC0467	OQ174613
	MHNC-UP-AMP 392	IAHTP065-22	Príncipe Island: Biosphere Reserve, trail to Santa Joaquina overview [1.6048, 7.4018, 315 m]		OQ174602
	MHNC-UP-AMP 396	IAHTP064-22	Príncipe Island: Biosphere Reserve, trail to Santa Joaquina overview [1.6048, 7.4018, 315 m]		OQ174644
	MHNC-UP-AMP 395	IAHTP063-22	Príncipe Island: Biosphere Reserve, trail to Santa Joaquina overview [1.6048, 7.4018, 315 m]		OQ174620
	MHNC-UP-AMP 393	IAHTP062-22	Príncipe Island: Biosphere Reserve, trail to Santa Joaquina overview [1.6048, 7.4018, 315 m]		OQ174577
	MHNC-UP-AMP 398	IAHTP061-22	Príncipe Island: Biosphere Reserve, trail to Santa Joaquina overview [1.6048, 7.4018, 315 m]		OQ174650
***Hyperoliusmolleri* (Bedriaga, 1892)**	MHNC-UP-AMP 667	IAHTP052-22	São Tomé Island: Roça Santo António, surroundings [0.2362 , 6.7274, 71 m]	BOLD:AEU9947	OQ174614
	MHNC-UP-AMP 666	IAHTP051-22	São Tomé Island: Roça Santo António, surroundings [0.2362 , 6.7274, 71 m]		OQ174611
	MHNC-UP-AMP 665	IAHTP044-22	São Tomé Island: Bom Sucesso, plantation area, Botanical Garden surroundings [0.2884, 6.6118, 1400 m]		OQ174646
	MHNC-UP-AMP 660	IAHTP037-22	São Tomé Island: Botanical Garden surroundings, Bom Sucesso, dirt road to CST tower [0.2796, 6.6093, 1212 m]		OQ174649
	MHNC-UP-AMP 659	IAHTP036-22	Tomé Island: Botanical Garden surroundings, Bom Sucesso, dirt road to CST tower [0.8497, 6.6099, 1149 m]		OQ174587
	MHNC-UP-AMP 658	IAHTP034-22	Tomé Island: Botanical Garden surroundings, Bom Sucesso, dirt road to CST tower [0.8497, 6.6099, 1149 m]		OQ174643
***Hyperoliusthomensis* (Bocage, 1886)**	MHNC-UP-AMP 661	IAHTP039-22	São Tomé Island: CST tower, near Bom Sucesso [0.2759, 6.6057, 1325 m]	BOLD:AEU9948	OQ174586
**Family Phrynobatrachidae**					
**Genus *Phrynobatrachus***					
*Phrynobatrachusdispar* (Peters, 1870)*	MHNC-UP-AMP 399	IAHTP067-22	Príncipe Island: Biosphere Reserve, trail to Santa Joaquina overview [1.6048, 7.4018, 315	BOLD:ADC0190	OQ174590
	MHNC-UP-AMP 400	IAHTP066-22	Príncipe Island: Biosphere Reserve, trail to Santa Joaquina overview [1.6048, 7.4018, 315 m]		OQ174576
***Phrynobatrachusleveleve* Uyeda, Drewes & Zimkus, 2007**	MHNC-UP-AMP 664	IAHTP043-22	São Tomé Island: Bom Sucesso, plantation area, Botanical Garden surroundings [0.2884, 6.6118, 1155 m]	BOLD:AEV9460	OQ174639
	MHNC-UP-AMP 663	IAHTP042-22	São Tomé Island: Bom Sucesso, plantation area, Botanical Garden surroundings [0.2884, 6.6118, 1155 m]		OQ174638
	MHNC-UP-AMP 662	IAHTP041-22	São Tomé Island: Bom Sucesso, plantation area, Botanical Garden surroundings [0.2884, 6.6118, 1155 m]		OQ174634
**Family Ptychadenidae**					
**Genus *Ptychadena***					
*Ptychadenanewtoni* (Bocage, 1886)*	CAS 261041	IAHTP084-22	São Tomé Island: outside of Malanza village, EMOLVA plantation [0.1149, 6.5929, 121 m]	BOLD:AAX7206	OQ174608
**ORDER GYMNOPHIONA**					
**Family Dermophiidae**					
**Genus *Schistometopum***					
*Schistometopumephele* Taylor, 1965*	MHNC-UP-AMP 673	IAHTP057-22	São Tomé Island: Água-Izé [0.2180, 6.7251, 47 m]	BOLD:AAN0016	OQ174591
***Schistometopumthomense* (Bocage, 1873)**	MHNC-UP-AMP 391	IAHTP027-22	São Tomé Island: Obô National Park, Botanical Garden, Bom Sucesso [0.28886, 6.6124, 1155 m]	BOLD:AEU6240	OQ174647
	MHNC-UP-AMP 675	IAHTP059-22	São Tomé Island: Água-Izé [0.2180, 6.7251, 47 m]		OQ174605
	MHNC-UP-AMP 674	IAHTP058-22	São Tomé Island: Água-Izé [0.2180, 6.7251, 47 m]		OQ174625
	MHNC-UP-AMP 672	IAHTP056-22	São Tomé Island: Roça Santo António, surroundings [0.2362, 6.7274, 71 m]		OQ174584
	MHNC-UP-AMP 671	IAHTP055-22	São Tomé Island: Roça Santo António, surroundings [0.2362, 6.7274, 71 m]		OQ174622
**REPTILIA**					
**ORDER SQUAMATA**					
**Family Gekkonidae**					
**Genus *Hemidactylus***					
***Hemidactylusgreeffii* Bocage, 1886**	MHNC-UP-REP 906	IAHTP031-22	São Tomé Island: Anambó, Padrão dos Descobrimentos [0.3251, 6.5093, 88 m]	BOLD:AEV3106	OQ174597
***Hemidactylusgreeffii* Bocage, 1886**	MHNC-UP-REP 908	IAHTP033-22	São Tomé Island: Anambó, Padrão dos Descobrimentos [0.3251, 6.5093, 88 m]	BOLD:AEV3106	OQ174630
	MHNC-UP-REP 907	IAHTP032-22	São Tomé Island: Anambó, Padrão dos Descobrimentos [0.3251, 6.5093, 88 m]		OQ174617
***Hemidactyluslongicephalus* Bocage, 1873**	CAS 218939	IAHTP082-22	São Tomé Island: coast road, SW of Lagoa Azul [0.4045, 6.6098, 18 m]	BOLD:AEW3810	OQ174651
	MHNC-UP-REP 911	IAHTP040-22	São Tomé Island: cistern, Botanical Garden surroundings, Bom Sucesso [0.2884, 6.6118, 1155 m]	BOLD:AEW3809	OQ174589
*Hemidactylusmabouia* (Moreau de Jonnès, 1818)*	MHNC-UP-REP 915	IAHTP047-22	São Tomé Island: São Tomé city, on a wall [0.3428, 6.7386, 10 m]	BOLD:ADI2267	OQ174618
***Hemidactylusprincipensis* Miller, Sellas & Drewes, 2012**	MHNC-UP-REP 853	IAHTP021-22	Príncipe Island: trail to Santo Cristo [1.6330, 7.4281, 157 m]	BOLD:AEW0476	OQ174653
	MB03-001014	IAHTP012-22	Tinhosa Grande Islet [1.3433, 7.2916, 61 m]		OQ174581
	MB03-001013	IAHTP011-22	Tinhosa Grande Islet [1.3439, 7.2926, 47 m]		OQ174648
	MB03-001011	IAHTP010-22	Tinhosa Grande Islet [1.3439, 7.2926, 47 m]		OQ174640
**Genus *Lygodactylus***					
***Lygodactylusdelicatus* Peters, 1881**	MHNC-UP-REP 857	IAHTP024-22	Príncipe Island: Porto Real, hospital ruins [1.6221, 7.4038, 137 m]	BOLD:AEV6848	OQ174642
***Lygodactylusthomensis* (Peters, 1881)**	MHNC-UP-REP 905	IAHTP030-22	São Tomé Island: Santana beach [0.2452, 6.7452, 23 m]	BOLD:AEW0905	OQ174635
	MHNC-UP-REP 904	IAHTP029-22	São Tomé Island: Santana beach [0.2452, 6.7452, 23 m]		OQ174641
**Family Scincidae**					
**Genus *Feylinia***					
***Feyliniapolylepis* Bocage, 1887**	MHNC-UP-REP 856	IAHTP023-22	Príncipe Island: Porto Real surroundings [1.6237, 7.4066, 126 m]	BOLD:AEV2384	OQ174578
	MHNC-UP-REP 847	IAHTP073-22	Príncipe Island: Porto Real surroundings [1.6237, 7.4066, 126 m]		OQ174583
	MHNC-UP-REP 846	IAHTP072-22	Príncipe Island: Porto Real surroundings [1.6237, 7.4066, 126 m]		OQ174623
	MHNC-UP-REP 845	IAHTP071-22	Príncipe Island: Porto Real surroundings [1.6237, 7.4066, 126 m]		OQ174585
	MHNC-UP-REP 844	IAHTP070-22	Príncipe Island: Biosphere Reserve, trail to Santa Joaquina overview [1.6048, 7.4018, 315 m]		OQ174612
	MHNC-UP-REP 843	IAHTP069-22	Príncipe Island: Biosphere Reserve, trail to Santa Joaquina overview [1.6048, 7.4018, 315 m]		OQ174633
**Genus *Panaspis***					
***Panaspisafricana* (Gray, 1845)**	MHNC-UP-REP 854	IAHTP022-22	Príncipe Island: trail to Santo Cristo [1.6330, 7.4281, 157 m]	BOLD:AEU9662	OQ174596
	MHNC-UP-REP 849	IAHTP075-22	Príncipe Island: Biosphere Reserve, trail to Santa Joaquina overview [1.6048, 7.4018, 315 m]		OQ174645
***Panaspisthomensis* Ceríaco, Soares, Marques, Bastos-Silveira, Scheinberg, Harris, Brehm & Jesus in Soares, Ceríaco, Marques, Bastos-Silveira, Scheinberg, Harris, Brehm & Jesus, 2018**	MHNC-UP-REP 840	IAHTP018-22	São Tomé Island: Obô National Park, Botanical Garden, Bom Sucesso [0.2888, 6.6124, 1155 m]	BOLD:AEU9663	OQ174579
	MHNC-UP-REP 839	IAHTP017-22	São Tomé Island: Obô National Park, Botanical Garden, Bom Sucesso [0.2888, 6.6124, 1155 m]		OQ174619
	MHNC-UP-REP 912	IAHTP045-22	São Tomé Island: trail to Lagoa Amélia [0.2887, 6.6105, 1163 m]		OQ174601
	MHNC-UP-REP 909	IAHTP035-22	São Tomé Island: Obô National Park, Botanical Garden, Bom Sucesso [0.2888, 6.6124, 1155 m]		OQ174582
**Genus *Trachylepis***					
***Trachylepisadamastor* Ceríaco, 2015**	MB03-001050	IAHTP009-22	Tinhosa Grande Islet [1.3424, 7.2890, 41 m]	BOLD:AEU9663	OQ174579
***Trachylepisadamastor* Ceríaco, 2015**	MB03-001049	IAHTP008-22	Tinhosa Grande Islet [1.3424, 7.2890, 41 m]	BOLD:AEU9663	OQ174629
	MB03-001048	IAHTP007-22	Tinhosa Grande Islet [1.3427, 7.2914, 55 m]		OQ174603
	MB03-001047	IAHTP006-22	Tinhosa Grande Islet [1.3431, 7.2917, 60 m]		OQ174580
	MB03-001046	IAHTP005-22	Tinhosa Grande Islet [1.3436, 7.2922, 40 m]		OQ174600
	MB03-001045	IAHTP004-22	Tinhosa Grande Islet [1.3437, 7.2924, 35 m]		OQ174615
	MB03-001044	IAHTP003-22	Tinhosa Grande Islet [1.3438, 7.2926, 30 m]		OQ174621
	MB03-001043	IAHTP002-22	Tinhosa Grande Islet [1.3414, 7.2932, 64 m]		OQ174588
	MHNC-UP-REP 851	IAHTP077-22	Príncipe Island: trail to Santo Cristo [1. 6330, 7.4281, 157 m]		OQ174609
	MHNC-UP-REP 848	IAHTP074-22	Príncipe Island: Porto Real surroundings [1.6237, 7.4066, 126 m]		OQ174632
***Trachylepisaffinis* (Gray, 1838)**	MHNC-UP-REP 858	IAHTP025-22	Príncipe Island: Banana beach overview [1.6884, 7.4435, 99 m]	BOLD:AEW1901	OQ174592
***Trachylepisthomensis* Ceríaco, Marques & Bauer, 2016**	MHNC-UP-REP 842	IAHTP020-22	São Tomé Island: Escola Portuguesa de São Tomé e Príncipe [0.3543, 6.7186, 42 m]	BOLD:AEU7392	OQ174626
	MHNC-UP-REP 841	IAHTP019-22	São Tomé Island: Escola Portuguesa de São Tomé e Príncipe [0.3546, 6.7185, 38 m]		OQ174607
**Family Typhlopidae**					
**Genus *Afrotyphlops***					
***Afrotyphlopselegans* (Peters 1868)**	MB03-000969	IAHTP016-22	Príncipe Island: Porto Real [1.6243, 7.4053, 125 m]	BOLD:AEV9368	OQ174636
**Genus *Letheobia***					
***Letheobiafeae* (Boulenger, 1906)**	CAS 218907	IAHTP080-22	São Tomé Island: on road between Bombaim and Santa Adelaide at rio Abade bridge [0.2542, 6.6300, 1261 m]	BOLD:AEW5328	OQ174610
***Letheobianewtoni* (Bocage, 1890)**	MB03-000974	IAHTP001-22	São Tomé Island: Botanical Garden, Bom Sucesso [0.2743, 6.5858, 1156 m]	BOLD:AEV5663	OQ174637
	CAS 218908	IAHTP081-22	São Tomé Island: on road between Bombaim and Santa Adelaide at rio Abade bridge [0.2542, 6.6300, 1261 m]	BOLD:AEV5664	OQ174599
**Family Colubridae**					
**Genus *Hapsidophrys***					
***Hapsidophrysprincipis* (Boulenger, 1906)**	MHNC-UP-REP 859	IAHTP026-22	Príncipe Island: Road to Bom Bom resort [1.6885, 7.4039, 43 m]	BOLD:AEW0890	OQ174593
**Genus *Philothamnus***					
***Philothamnusthomensis* Bocage, 1882**	CAS 233675	IAHTP083-22	São Tomé Island: bridge at Água Panada near Santa Catarina [0.2680, 6.6489, 418 m]	BOLD:AEV9763	OQ174652
	CAS 218823	IAHTP079-22	São Tomé Island: mouth of Água Anambó [0.3257, 6.5084, 14 m]		OQ174575
**Family Lamprophiidae**					
**Genus *Boaedon***					
***Boaedonbedriagae* Boulenger, 1906**	MHNC-UP-REP 917	IAHTP049-22	São Tomé Island: on a dirt road next to the cocoa plantation, on the outskirts of Roça Santo António [00.2365, 6.7275, 71 m]	BOLD:AEW1645	OQ174624
***Boaedonmendesi* Ceríaco, Arellano, Jadin, Marques, Parrinha & Hallermann, 2021**	MHNC-UP-REP 850	IAHTP076-22	Príncipe Island: Biosphere Reserve, tril to Santa Joaquina overview [1.6048, 7.4018, 315 m]	BOLD:AEW1644	OQ174616
**Family Elapidae**					
**Genus *Naja***					
***Najaperoescobari* Ceríaco, Marques, Schmitz & Bauer, 2017**	MHNC-UP-REP 913	IAHTP046-22	São Tomé Island: trail to Lagoa Amélia [0.2717, 6.6280, 967 m]	BOLD:AEU9514	OQ174594
**ORDER TESTUDINES**					
**Family Pelomedusidae**					
**Genus *Pelusios***					
*Pelusioscastaneus* (Schweigger, 1812)*	MHNC-UP-REP 919	IAHTP060-22	São Tomé Island: Roça Santo António surroundings, in a small stream [0.2392, 6.7305, 64 m]	BOLD:AAX1351	OQ174606

### ﻿Taxonomic allocation

The allocation of the collected specimens to the correct taxon followed the most updated taxonomic bibliography available for each group. This bibliography includes both morphological and molecular data and provides the most updated information regarding the occurring taxa. In many cases, the specimens used to generate the reference DNA barcodes in our study were those also used in some of these taxonomic revisions (e.g., Ceríaco et al. 2022). In all cases, we consulted the original description of the taxon and, whenever possible, examined the extant type specimens.

For the genus *Hyperolius* we followed Bell (2016) and Bell and Irian (2019), while for the genus *Phrynobatrachus* we followed Uyeda et al. (2007). Bell et al. (2015) and Bell and Irian (2019) noted that *Hyperoliusthomensis* (Bocage, 1886), and *H.molleri* (Bedriaga, 1892), both endemic to São Tomé Island, hybridize where their ranges meet. Regarding the endemic caecilians of the genus *Schistometopum*, we followed the recent revision of O’Connell et al. (2021), which supported the existence of two separate species in São Tomé Island, distinguished both morphologically and molecularly. Similarly to the case of São Tomé Island’s *Hyperolius*, the two *Schistometopum* species are also known to hybridize (O’Connell et al. 2021). The systematics of the Príncipe Island endemic *Leptopelispalmatus* was recently studied by Jaynes et al. (2021) and being the only representative of the genus in the country, it is an easily diagnosable species with respect to the remaining batrachofauna. Similarly, the São Tomé Island endemic *Ptychadenanewtoni* is the single species of the genus occurring in the Island and poses no morphological identification issues, and Measey et al. (2007) assessed its systematic placement.

Regarding the terrestrial reptiles, the members of the genus *Trachylepis* (family Scincidae) have been extensively reviewed by Ceríaco (2015), and Ceríaco et al. (2016, 2020a), while those of the genus *Panaspis* (family Scincidae) have been critically addressed by Soares et al. (2018). The remaining member of family Scincidae, the Príncipe endemic *Feyliniapolylepis*, has a stable taxonomic history, since the major review of the group by Brygoo and Roux-Estève (1983). Geckos of the genus *Lygodactylus* have been reviewed by Pasteur (1962), who pointed out the morphological differences between the two island’s populations, considering them to be two different subspecies. Molecular support for this split was provided by Jesus et al. (2006), and each island population is considered as a separate species by Ceríaco et al. (2018, 2022). The taxonomy, phylogenetic affinities and nomenclatural history of the species of the genus *Hemidactylus* have been addressed by Miller et al. (2012) and Ceríaco et al. (2020b).

The main taxonomic uncertainties still open in the São Tomé and Príncipe herpetofauna lie within the scolecophidian snakes, namely those of the genus *Letheobia*. Four different taxa have been described so far: *Letheobiafeae* and *L.newtoni* from São Tomé Island, and *L.principis* and *L.naveli* from Príncipe Island. The two species from Príncipe Island were synonymized respectively with those from São Tomé Island by Roux-Estève (1974) based on morphological characters. No molecular data exist for the Príncipe populations, and thus their taxonomic relationships with the São Tomé forms have not been fully ascertained (Ceríaco et al. 2022). Given the patterns of speciation in the archipelago and the morphological conservatism of these snakes, the possibility that the Príncipe forms represent valid species needs to be investigated (Ceríaco et al. 2022). Given this uncertainty, we conservatively follow Roux-Estève (1974) and consider *L.principis* and *L.naveli* as junior synonyms of *L.feae* and *L.newtoni*, respectively. The other occurring scolecophidian snake, the Príncipe endemic *Afrotyphlopselegans*, is the only representative of the genus in the country and it is easily diagnosable against the remaining snakes. It was placed in the context of a global phylogeny by Hedges et al. (2014). Within the remaining snake groups, the species of the genus *Boaedon* (family Lamprophiidae) have been taxonomically reviewed by Ceríaco et al. (2021b), while the colubrids, genera *Philothamnus* and *Hapsidophrys*, have a very stable taxonomical history, with recent studies supporting their taxonomic identity (Engelbrecht et al. 2019; Jesus et al. 2009, respectively). The only confirmed species of elapid snake, *Najaperoescobari*, endemic to São Tomé Island, has been recently reviewed by Ceríaco et al. (2017). While a putative species of green mamba (genus *Dendroaspis*) has been cited from São Tomé Island (Ceríaco et al. 2018, 2022), its occurrence could not be confirmed and is therefore not considered here. Finally, the only terrapin in the country, *Pelusioscastaneus*, has been confirmed to belong to the nominotypical form through molecular data (Fritz et al. 2010; Kindler et al. 2016). Roaming crocodilians, such as the recent arrival of a living individual of *Crocodylusniloticus* to the beaches of southeastern São Tomé Island, or non-established invasive species occasionally arriving to these islands (see Ceríaco et al. 2022), are not covered in this paper. Sea turtles are also not covered, as they are comprehensively treated elsewhere (Vargas et al. 2009).

### ﻿DNA extraction, amplification, and sequencing

Genomic DNA was extracted from liver tissue sample using the EasySpin Genomic DNA Tissue Kit (Citomed) according to the manufacturer’s protocol. DNA amplification was performed using two different primer pairs, that amplify partially overlapping fragments (LC + BH) of the 658 bp barcoding region of the Cytochrome c oxidase subunit I - COI mitochondrial gene (Folmer et al. 1994). We used the primers FwhF1 (Vamos et al. 2017) + C_R (Shokralla et al. 2015) for LC, and BF3 (Elbrecht et al. 2019) + BR2 (Elbrecht and Leese 2017) for BH amplification. Primers were ordered with 5’ adaptor sequences to ensure they were compatible with downstream indexing allowing for a two-step PCR protocol. First-round PCRs were performed in 10 µl reactions, containing 5 µl of Multiplex PCR Master Mix (Qiagen, Germany), 0.3 µl of each 10 mM primer, and 1–2 µl of DNA, with the remaining volume in water. PCR cycling conditions consisted in an initial denaturation at 95 °C for 15 min, followed by 45 cycles of denaturation at 95 °C for 30 sec, annealing at 45 °C for 45 sec, and extension at 72 °C for 45 sec, and a final elongation step at 60 °C for 10 min. Successful amplification was validated through 2% agarose gel electrophoresis and samples selected for sequencing followed for a second PCR, where Illumina P5 and P7 adapters with custom 7 bp long barcodes were attached to each PCR product. The index PCR was performed in a volume of 10 µl, including 5 µL of KAPA HiFi PCR Kit (KAPA Biosystems, U.S.A.), 0.5 µl of each 10 mM indexing primer, and 2 µl of diluted PCR product (usually 1:4). PCR cycling conditions were as before, except that only 10 cycles were performed and at an annealing temperature of 55 °C. The amplicons were purified using AMPure XP beads (Beckman Coulter, U.S.A.) and quantified using NanoDrop 1000 (Thermo Scientific, U.S.A.). Clean PCR products were then pooled equimolarly per fragment. Each pool was quantified with KAPA Library Quantification Kit Illumina Platforms (KAPA Biosystems, U.S.A.) and the 2200 Tapestation System (Agilent Technologies, California, USA) was used for fragment length analysis prior to sequencing (Paupério et al. 2018). DNA sequencing was done at CIBIO (Centro de Investigação em Biodiversidade e Recursos Genéticos) facilities on an Illumina MiSeq benchtop system, using a V2 MiSeq sequencing kit (2× 250 bp).

### ﻿Bioinformatics processing and data analysis

Illumina sequencing reads were processed using OBITools (Boyer et al. 2015) and VSEARCH (Rognes et al. 2016). Briefly, paired-end reads were aligned, collapsed into exact sequence variants, filtered by length, denoised, and checked for chimeras. The resulting sequences from both LC and BH fragments of each sample were further assembled using CAP3 (Huang and Madan 1999) to produce a single 658 bp contig per sample. All sequences in the dataset were submitted to Barcode of Life Data System (BOLD) and GenBank databases and, to each sequenced specimen, the morphological identification was contrasted with the results of the BLAST of the newly-generated DNA barcodes in the BOLD Identification Engine. Barcode Index Numbers (BIN) clusters were retrieved from BIN algorithm implemented in BOLD SYSTEMS. The BOLDBIN system uses algorithms to cluster sequences into operational taxonomic units (OTUs) that closely correspond to species (Ratnasingham and Hebert 2007, 2013). Interspecific distances were calculated using MEGA11 (Tamura et al. 2021).

## ﻿Results

### ﻿Morphological Identification

The 18 taxa (six amphibians, 12 terrestrial reptiles) occurring in São Tomé Island and its surrounding islets of Rolas, Cabras, and Santana, as well as the 17 taxa (three amphibians, 14 terrestrial reptiles) occurring in Príncipe Island and its surrounding islets of Tinhosa Grande and Joquéi are easily distinguishable from each other by a set of morphological, meristic, coloration and ecological characters (see Keys below).

### ﻿Illustrated key to the species of amphibians and terrestrial reptiles from São Tomé Island, Rolas, Cabras, and Santana islets

**Table:** 

1	Skin smooth, not covered with scales (Fig. 1A)	**2 (Class Amphibia)**
–	Skin covered with scales (Fig. 1B)	**7 (Class Reptilia)**
2	**Class Amphibia** Absence of limbs (Fig. 2A)	**3 (Order Gymnophiona)**
–	**Class Amphibia** Four limbs present (Fig. 2B)	**4 (Order Anura)**
3	**Class Amphibia: Order Gymnophiona** Immaculate bright yellow skin (Fig. 3A)	***Schistometopumthomense***
–	**Class Amphibia: Order Gymnophiona** Bright yellow skin flecked with brown markings (Fig. 3B)	***Schistometopumephele***
4	**Class Amphibia: Order Anura** Adhesive terminal discs on fingers and toes (Fig. 4A)	**5 (Genus *Hyperolius*)**
–	**Class Amphibia: Order Anura** No adhesive terminal discs on fingers and toes (Fig. 4B)	**6 (Genera *Ptychadena* and *Phrynobatrachus*)**
5	**Class Amphibia: Order Anura: Genus *Hyperolius*** Snout as long as the eye diameter, finger, and toe disks orange above, male throat (Fig. 5A)	***Hyperoliusthomensis***
–	**Class Amphibia: Order Anura: Genus *Hyperolius*** Snout longer than the eye diameter, finger, and toe disks red above, male throat orange (Fig. 5B)	***Hyperoliusmolleri***
6	**Class Amphibia: Order Anura: Genera *Ptychadena*** and ***Phrynobatrachus*** Large animals (maximum snout-vent length 86 mm), acuminate snout (Fig. 6A), tympanum visible, presence of dorsal skin folds, presence of well-developed foot webbing (Fig. 6C)	***Ptychadenanewtoni***
–	**Class Amphibia: Order Anura: Genera *Ptychadena*** and ***Phrynobatrachus*** Small animals (maximum snout-vent length 21 mm), rounded snout (Fig. 6B), tympanum not visible, presence of dorsal warts, rudimentary foot webbing (Fig. 6D)	***Phrynobatrachusleveleve***
7	**Class Reptilia** Presence of a bony shell (Fig. 7A)	***Pelusioscastaneus***
–	**Class Reptilia** Absence of a bony shell (Fig. 7B)	**8 (Order Squamata)**
8	**Class Reptilia, Order Squamata** Presence of four limbs (Fig. 8A)	**9 (Suborder Sauria)**
–	**Class Reptilia, Order Squamata** Absence of four limbs (Fig. 8B)	**14 (Suborder Serpentes)**
9	**Class Reptilia, Order Squamata, Suborder Sauria** Presence of toepads on the ventral area of the digits (Fig. 9A), skin comprising granular scales with or without enlarged tubercles (Fig. 9C), eyes large	**10 (Family Gekkonidae)**
–	**Class Reptilia, Order Squamata, Suborder Sauria** Presence of lamellae on the ventral area of the digits (Fig. 9B), skin comprising overlapping cycloid keeled scales (Fig. 9D), eyes small	**13 (Family Scincidae)**
10	**Class Reptilia, Order Squamata, Suborder Sauria, Family Gekkonidae** First toe rudimentary (Fig. 10A), pupils round (Fig. 10C), diurnal, slender animal, green to dark brown	***Lygodactylusthomensis***
–	**Class Reptilia, Order Squamata, Suborder Sauria, Family Gekkonidae** First toe well developed (Fig. 10B), pupils vertical (Fig. 10D), nocturnal, robust animal, whitish to brown	**11 (Genus *Hemidactylus*)**
11	**Class Reptilia, Order Squamata, Suborder Sauria, Family Gekkonidae, Genus *Hemidactylus*** Absence of terminal phalanx and claw on first digit (Fig. 11A)	***Hemidactylusgreeffii***
–	**Class Reptilia, Order Squamata, Suborder Sauria, Family Gekkonidae, Genus *Hemidactylus*** Presence of terminal phalanx and claw on first digit (Fig. 11B)	**12**
12	**Class Reptilia, Order Squamata, Suborder Sauria, Family Gekkonidae, Genus *Hemidactylus*** Median subcaudals broadened transversely (> ½ tail width; Fig. 12A), presence of 28–39 precloacal femoral pores in males (Fig. 12C)	***Hemidactylusmabouia***
–	**Class Reptilia, Order Squamata, Suborder Sauria, Family Gekkonidae, Genus *Hemidactylus*** Median subcaudals small (< ½ tail width; Fig. 12B), presence of 4–11 precloacal-femoral pores in males (Fig. 12D)	***Hemidactyluslongicephalus***
13	**Class Reptilia, Order Squamata, Suborder Sauria, Family Scincidae** Dorsal scales smooth (Fig. 13A), small limbs and digits, small animal (max SVL 47.7 mm) (Fig. 13C)	***Panaspisthomensis***
–	**Class Reptilia, Order Squamata, Suborder Sauria, Family Scincidae** Dorsal scales keeled (Fig. 13B), well-developed limbs and digits, large animal (max SVL 98.2 mm) (Fig. 13D)	***Trachylepisthomensis***
14	**Class Reptilia, Order Squamata, Suborder Serpentes** Eyes rudimentary to non-visible (Fig. 14A), body with indistinct head, beaked snout dominated by very wide rostral scale (Fig. 14C), worm-like body shape	**15 (Family Typhlopidae)**
–	**Class Reptilia, Order Squamata, Suborder Serpentes** Eyes well developed and visible (Fig. 14B), body with distinct head, blunt snout with several cephalic scales of different sizes (Fig. 14D), snake-like body shape	**16 (Families Colubridae, Elapidae and Lamprophiidae)**
15	**Class Reptilia, Order Squamata, Suborder Serpentes, Family Typhlopidae** 26–28 midbody scale rows, rostral moderately acuminate (Fig. 15A)	***Letheobianewtoni***
–	**Class Reptilia, Order Squamata, Suborder Serpentes, Family Typhlopidae** 21–22 midbody scale rows, rostral extremely acuminate (Fig. 15B)	***Letheobiafeae***
16	**Class Reptilia, Order Squamata, Suborder Serpentes, Families Colubridae**, **Elapidae** and **Lamprophiidae** 15 midbody scale rows, slender snake, green, anal scale divided (Fig. 16A)	***Philothamnusthomensis***
–	**Class Reptilia, Order Squamata, Suborder Serpentes, Families Colubridae**, **Elapidae** and **Lamprophiidae** 19 or more midbody scale rows, robust snake, not green, anal scale un-divided (Fig. 16B)	**17**
17	**Class Reptilia, Order Squamata, Suborder Serpentes, Families Colubridae**, **Elapidae** and **Lamprophiidae** Dorsal coloration uniformly black, presence of specialized venom injecting fangs, presence of spike on the terminal part of the tail (Fig. 17A)	**Naja (Boulengerina) peroescobari**
–	**Class Reptilia, Order Squamata, Suborder Serpentes, Families Colubridae**, **Elapidae** and **Lamprophiidae** Dorsal coloration brownish with dorsolateral cream stripes, absence of specialized venom injecting fangs, absence of spike on the terminal part of the tail (Fig. 17B)	***Boaedonbedriagae***

### ﻿Illustrated key to the species of amphibians and terrestrial reptiles from Príncipe Island and Bombom, Joquéi, and Tinhosas islets

**Table:** 

18	Skin smooth, not covered with scales (Fig. 18A)	**19 (Class Amphibia)**
–	Skin covered with scales (Fig. 18B)	**21 (Class Reptilia)**
19	**Class Amphibia, Class Amphibia, Order Anura** No adhesive terminal discs on fingers and toes (Fig. 19A)	***Phrynobatrachusdispar***
–	**Class Amphibia, Class Amphibia, Order Anura** Adhesive terminal discs on fingers and toes (Fig. 19B)	**20 (Genera *Hyperolius* and *Leptopelis*)**
20	**Class Amphibia, Order Anura, Genus *Hyperolius*** Large animals (max SVL 110 mm), pupils vertical, eyes deep red, tympanum visible (Fig. 20A)	***Leptopelispalmatus***
–	**Class Amphibia, Order Anura, Genus *Hyperolius*** Small animals (max SVL 33 mm), pupils horizontal, eyes golden, tympanum not visible (Fig. 20B)	***Hyperoliusdrewesi***
21	**Class Reptilia** Presence of a bony shell (Fig. 21A)	***Pelusioscastaneus***
–	**Class Reptilia** Absence of a bony shell (Fig. 21B)	**22**
22	**Class Reptilia, Order Squamata** Presence of four limbs (Fig. 22A)	**23**
–	**Class Reptilia, Order Squamata** Absence of four limbs (Fig. 22B)	**29**
23	**Class Reptilia, Order Squamata** Skin comprising granular scales with or without enlarged tubercles (Fig. 23A), eyes large, presence of toepads on the ventral area of the digits (Fig. 23C)	**24 (Family Gekkonidae)**
–	**Class Reptilia, Order Squamata** Skin comprising overlapping cycloid keeled scales (Fig. 23B), eyes small, presence of lamellae on the ventral area of the digits (Fig. 23D)	**27 (Family Scincidae [part])**
24	**Class Reptilia, Order Squamata, Suborder Sauria, Family Gekkonidae** First toe rudimentary (Fig. 24A), pupils round (Fig. 24C), diurnal, slender animal, green to dark brown	***Lygodactylusdelicatus***
–	**Class Reptilia, Order Squamata, Suborder Sauria, Family Gekkonidae** First toe well developed (Fig. 24B), pupils vertical (Fig. 24D), nocturnal, robust animal, whitish to brown	**25 (Genus *Hemidactylus*)**
25	**Class Reptilia, Order Squamata, Suborder Sauria, Family Gekkonidae, Genus *Hemidactylus*** Absence of terminal phalanx and claw on first digit (Fig. 25A)	***Hemidactylusprincipensis***
–	**Class Reptilia, Order Squamata, Suborder Sauria, Family Gekkonidae, Genus *Hemidactylus*** Presence of terminal phalanx and claw on first digit (Fig. 25B)	**26**
26	**Class Reptilia, Order Squamata, Suborder Sauria, Family Gekkonidae, Genus *Hemidactylus*** Median subcaudals broadened transversely (> ½ tail width; Fig. 26A), presence of 28–39 precloacal-femoral pores in males (Fig. 26C)	***Hemidactylusmabouia***
–	**Class Reptilia, Order Squamata, Suborder Sauria, Family Gekkonidae, Genus *Hemidactylus*** Median subcaudals small (< ½ tail width; Fig. 26B), presence of 4–11 precloacal-femoral pores in males (Fig. 26D)	***Hemidactyluslongicephalus***
27	**Class Reptilia, Order Squamata, Suborder Sauria, Family Gekkonidae, Genus *Hemidactylus*** Dorsal scales smooth (Fig. 27A), small limbs and digits (Fig. 27C), small animal (max SVL 42.5 mm)	***Panaspisafricana***
–	**Class Reptilia, Order Squamata, Suborder Sauria, Family Gekkonidae, Genus *Hemidactylus*** Dorsal scales keeled (Fig. 27B), well-developed limbs and digits (Fig. 27D), large animal (max SVL 112 mm)	**28 (Genus *Trachylepis*)**
28	**Class Reptilia, Order Squamata, Suborder Sauria, Family Scincidae, Genus *Trachylepis*** Absence of stripes, back uniformly greenish brown or dark, medium to large-sized animal (max SVL 58–112 mm) (Fig. 28A)	***Trachylepisadamastor***
–	**Class Reptilia, Order Squamata, Suborder Sauria, Family Scincidae, Genus *Trachylepis*** Presence of a white stripe on the lower part of the flanks, back uniformly brownish, small to medium-sized animal (SVL 39–71 mm) (Fig. 28B)	***Trachylepisaffinis***
29	**Class Reptilia, Order Squamata, Suborder Sauria, Family Scincidae** and **Suborder Serpentes** Eyes rudimentary to non-visible (Fig. 29A), worm-like body shape	**30**
–	**Class Reptilia, Order Squamata, Suborder Sauria, Family Scincidae** and **Suborder Serpentes** Eyes well developed and visible (Fig. 29B), snake-like body shape	**33 (Families Colubridae and Lamprophiidae)**
30	**Class Reptilia, Order Squamata, Suborder Sauria, Family Scincidae** and **Suborder Serpentes** Acuminate snout and rostral scale roundish (Fig. 30A)	***Feyliniapolylepis***
–	**Class Reptilia, Order Squamata, Suborder Sauria, Family Scincidae** and **Suborder Serpentes** Short head and rostral scale in the shape of a fingernail (Fig. 30B)	**31 (Family Typhlopidae)**
31	**Class Reptilia, Order Squamata, Suborder Serpentes, Family Typhlopidae** Yellow coloration with black stripes (Fig. 31A), thick body, presence of a spike at the posterior end of the tail (Fig. 31C)	***Afrotyphlopselegans***
–	**Class Reptilia, Order Squamata, Suborder Serpentes, Family Typhlopidae** Beige coloration without stripes (Fig. 31B), thin body, absence of a spike at the posterior end of the tail (Fig. 31D)	**32 (Genus *Letheobia*)**
32	**Class Reptilia, Order Squamata, Suborder Serpentes, Family Typhlopidae, Genus *Letheobia*** 26–28 midbody scale rows, rostral moderately acuminate (Fig. 32A)	***Letheobianewtoni***
–	**Class Reptilia, Order Squamata, Suborder Serpentes, Family Typhlopidae, Genus *Letheobia*** 21–22 midbody scale rows, rostral extremely acuminate (Fig. 32B)	***Letheobiafeae***
33	**Class Reptilia, Order Squamata, Suborder Serpentes, Families Colubridae and Lamprophiidae** Smooth dorsal scales, brown to dark brown coloration, 24–29 midbody scale rows (Fig. 33A)	***Boaedonmendesi***
–	**Class Reptilia, Order Squamata, Suborder Serpentes, Families Colubridae and Lamprophiidae** Strongly keeled dorsal scales, blueish green coloration, 15 midbody scale rows (Fig. 33B)	***Hapsidophrysprincipis***

**Figure 1.**
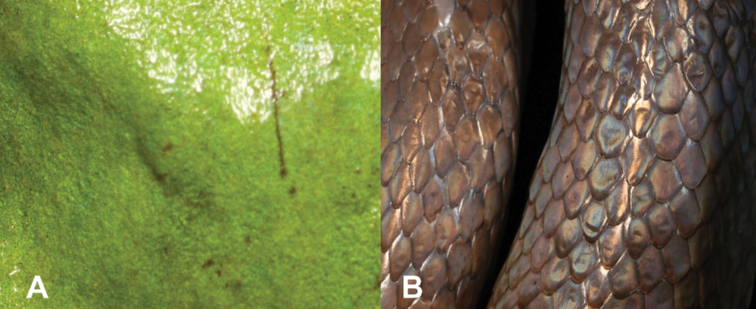
**A** smooth skin, typical of amphibians **B** skin covered with scales, typical of reptiles. Photographs by Luis M. P. Ceríaco.

**Figure 2.**
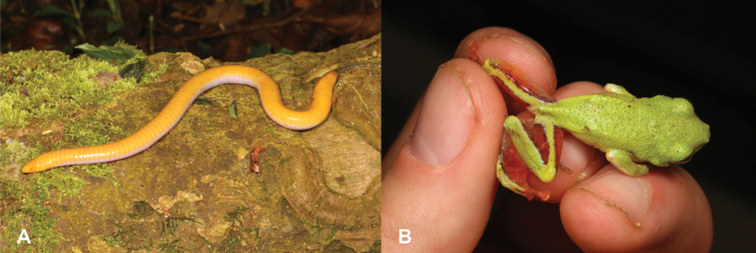
**A** absence of limbs, typical of order Gymnophiona**B** presence of four limbs, typical of order Anura.

**Figure 3.**
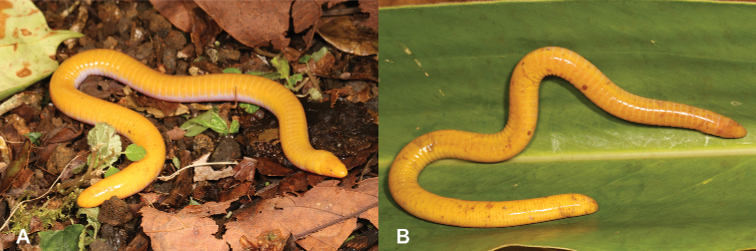
**A** immaculate bright yellow skin, typical of *S.thomense***B** bright yellow skin flecked with brown markings, typical of *S.ephele*. Photographs by Luis M. P. Ceríaco.

**Figure 4.**
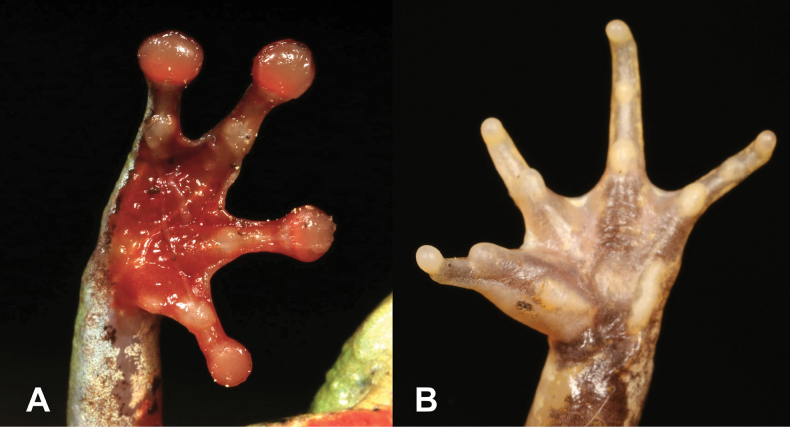
**A** adhesive terminal discs **B** no adhesive terminal discs. Photographs by Luis M. P. Ceríaco.

**Figure 5.**
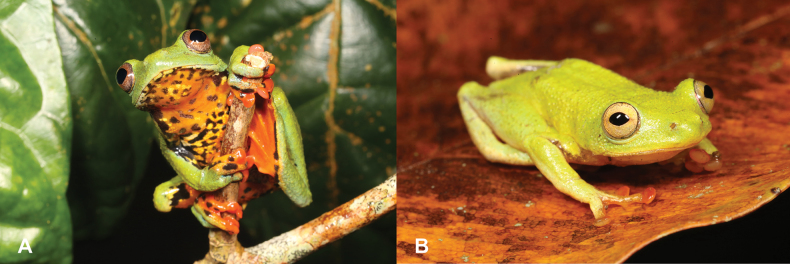
**A***Hyperoliusthomensis***B***Hyperoliusmolleri*. Photographs by Luis M. P. Ceríaco.

**Figure 6.**
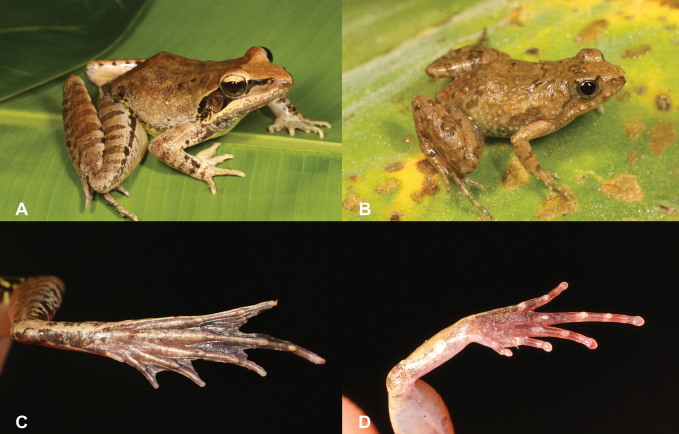
**A***Ptychadenanewtoni***B** foot with well-developed webbing **C***Phrynobatrachusleveleve***D** foot with rudimentary to no webbing. Photographs by Luis M. P. Ceríaco.

**Figure 7.**
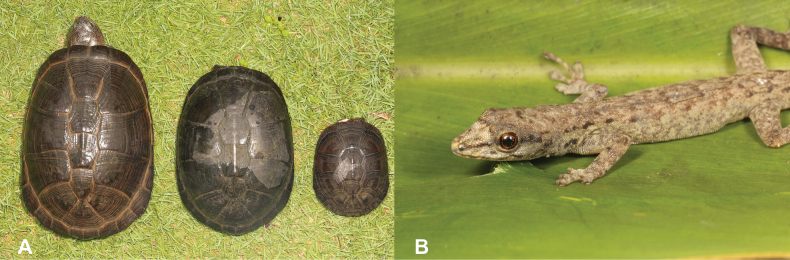
**A** presence of a bony shell, as typical of turtles, in this case *Pelusioscastaneus***B** absence of a bony shell, as typical of squamates. Photographs by Luis M. P. Ceríaco.

**Figure 8.**
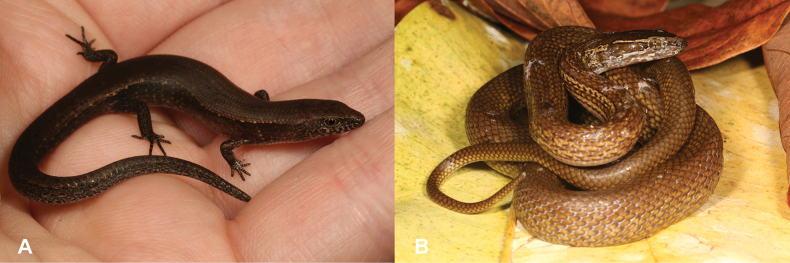
**A** presence of four limbs **B** absence of limbs. Photographs by Luis M. P. Ceríaco.

**Figure 9.**
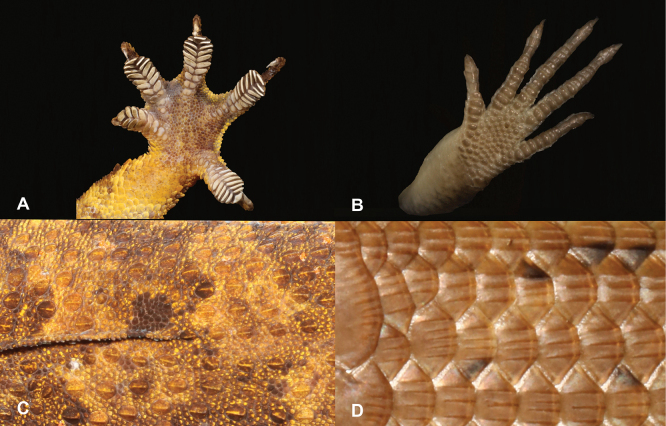
**A** presence of toepads on the ventral area of the digits **B** presence of lamellae on the ventral area of the digits **C** skin composed by granular scales with or without enlarged tubercles **D** skin composed by overlapping cycloid keeled scales. Photographs by Luis M. P. Ceríaco.

**Figure 10.**
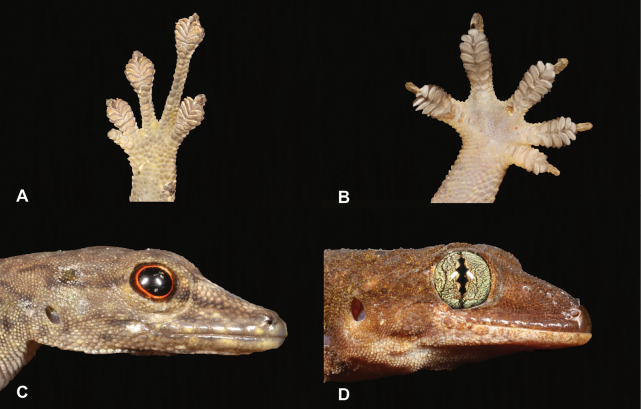
**A** first toe rudimentary **B** first toe well developed **C** pupils round **D** pupils vertical. Photographs by Luis M. P. Ceríaco.

**Figure 11.**
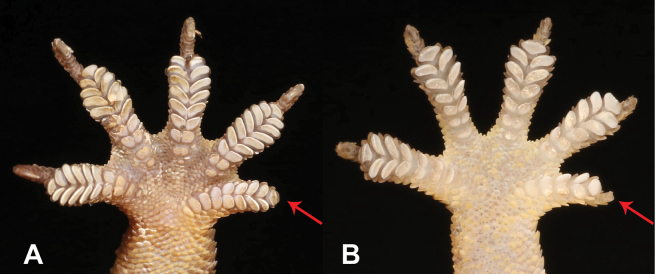
**A** absence of terminal phalanx and claw on first digit **B** presence of terminal phalanx and claw on first digit. Photographs by Luis M. P. Ceríaco.

**Figure 12.**
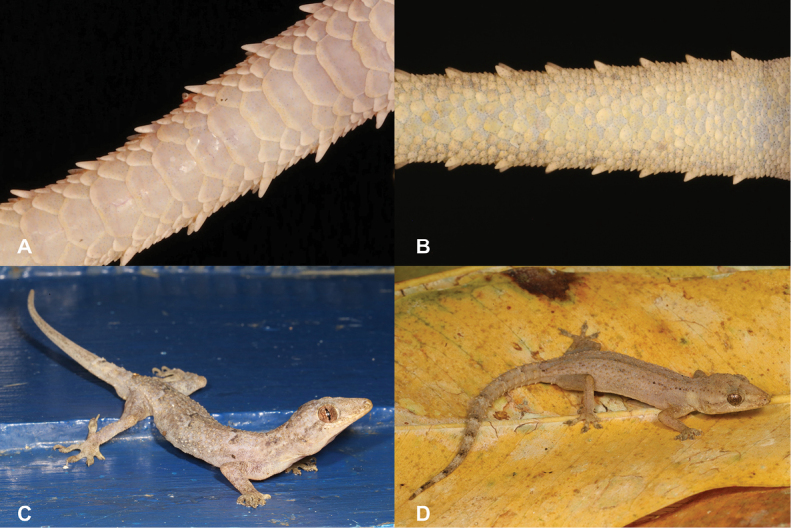
**A** median subcaudals broadened transversely **B** median subcaudals small **C***Hemidactylusmabouia***D***Hemidactyluslongicephalus*. Photographs by Luis M. P. Ceríaco.

**Figure 13.**
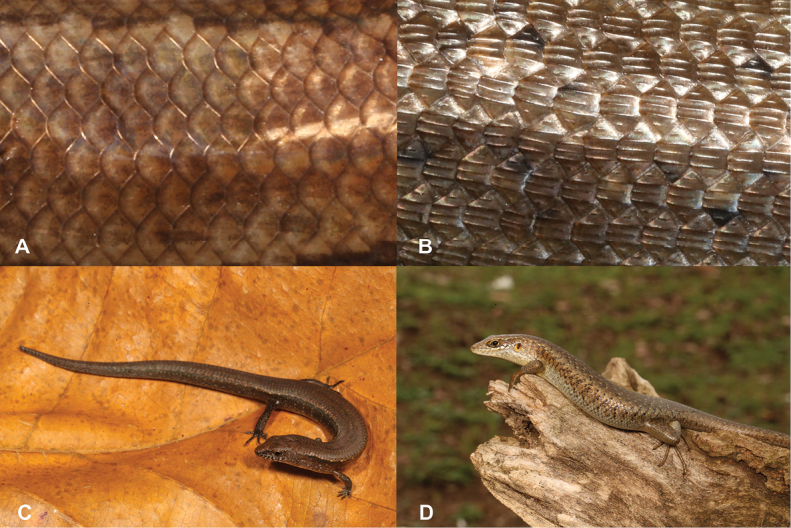
**A** dorsal scales smooth **B** dorsal scales keeled **C***Panaspisthomensis***D***Trachylepisthomensis*. Photographs by Luis M. P. Ceríaco.

**Figure 14.**
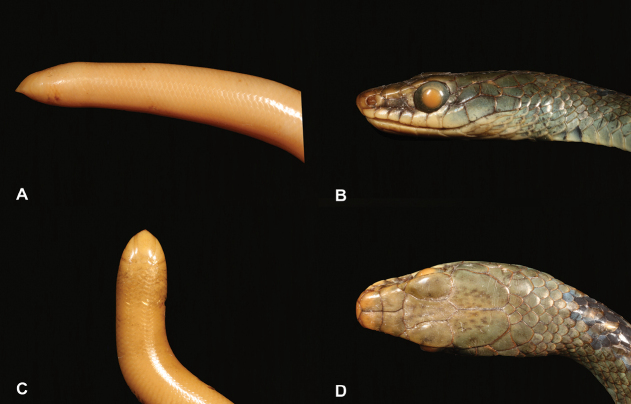
**A** eyes rudimentary to non-visible **B** eyes well developed and visible **C** body with indistinct head, beaked snout dominated by very wide rostral scale **D** body with distinct head, blunt snout with several cephalic scales of different sizes. Photographs by Luis M. P. Ceríaco.

**Figure 15.**
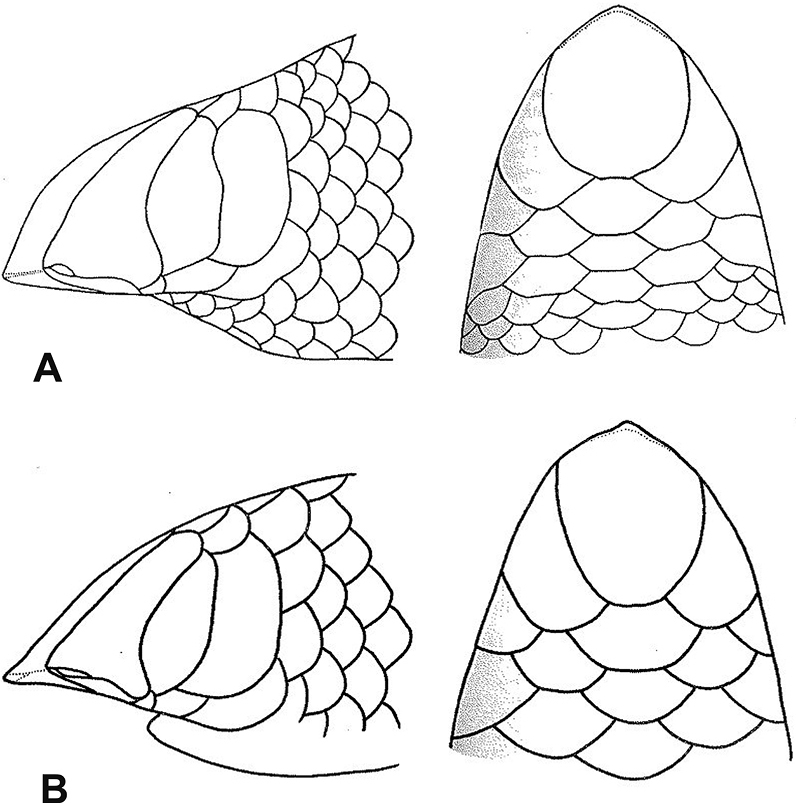
**A***Letheobianewtoni***B***Letheobiafeae* (adapted from Roux-Estève 1974). Photographs by Luis M. P. Ceríaco.

**Figure 16.**
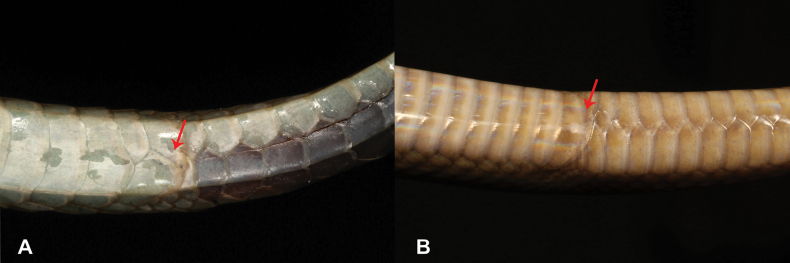
**A** anal scale divided **B** anal scale entire. Photographs by Luis M. P. Ceríaco.

**Figure 17.**
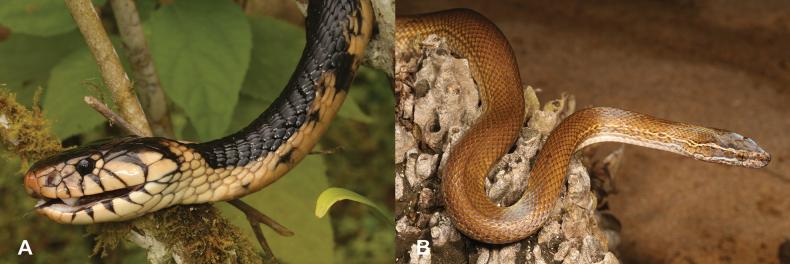
**A**Naja (Boulengerina) peroescobari**B***Boaedonbedriagae*. Photographs by Luis M. P. Ceríaco.

**Figure 18.**
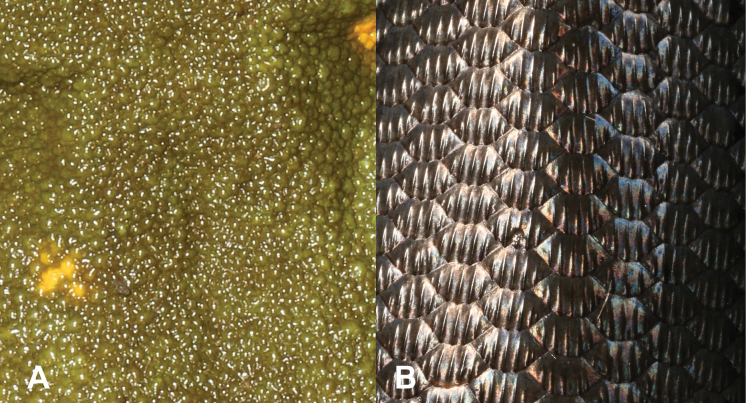
**A** smooth skin, typical of amphibians **B** skin covered with scales, typical of reptiles. Photographs by Luis M. P. Ceríaco.

**Figure 19.**
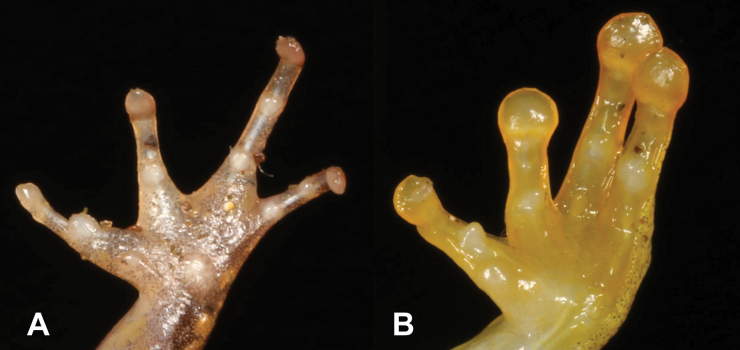
**A** no adhesive terminal disks **B** adhesive terminal disks. Photographs by Luis M. P. Ceríaco.

**Figure 20.**
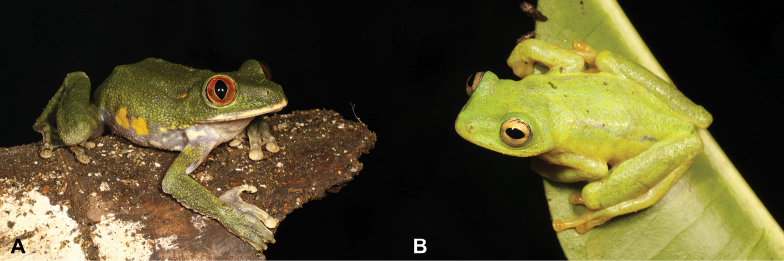
**A***Leptopelispalmatus***B***Hyperoliusdrewesi*. Photographs by Luis M. P. Ceríaco.

**Figure 21.**
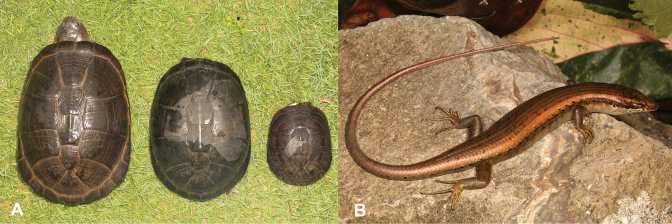
**A** presence of a bony shell, as typical of turtles, in this case *Pelusioscastaneus***B** absence of a bony shell, as typical of squamates. Photographs by Luis M. P. Ceríaco.

**Figure 22.**
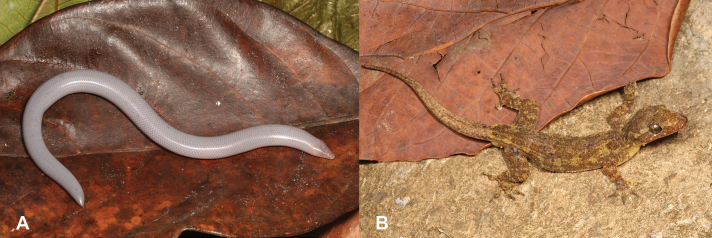
**A** absence of limbs **B** presence of four limbs. Photographs by Luis M. P. Ceríaco.

**Figure 23.**
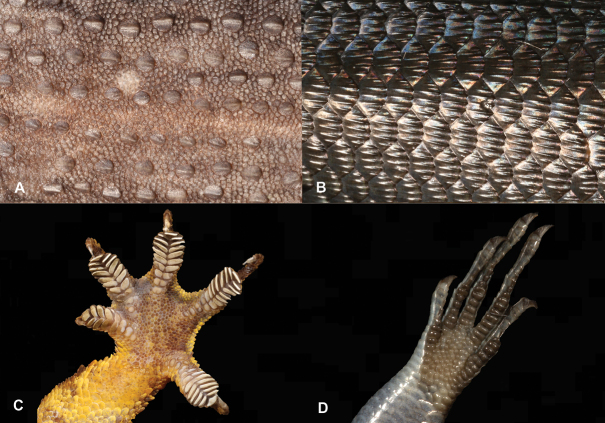
**A** skin composed by granular scales with or without enlarged tubercles **B** skin composed by overlapping cycloid keeled scales **C** presence of toepads on the ventral area of the digits **D** presence of lamellae on the ventral area of the digits. Photographs by Luis M. P. Ceríaco.

**Figure 24.**
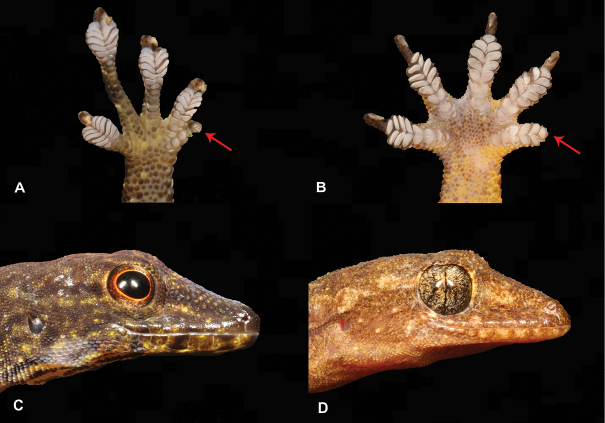
**A** first toe rudimentary **B** first toe well developed **C** pupils round **D** pupils vertical. Photographs by Luis M. P. Ceríaco.

**Figure 25.**
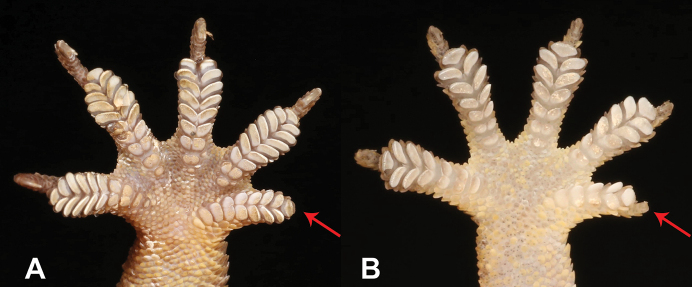
**A** absence of terminal phalanx and claw on first digit, *Hemidactylusprincipensis***B** presence of terminal phalanx and claw on first digit. Photographs by Luis M. P. Ceríaco.

**Figure 26.**
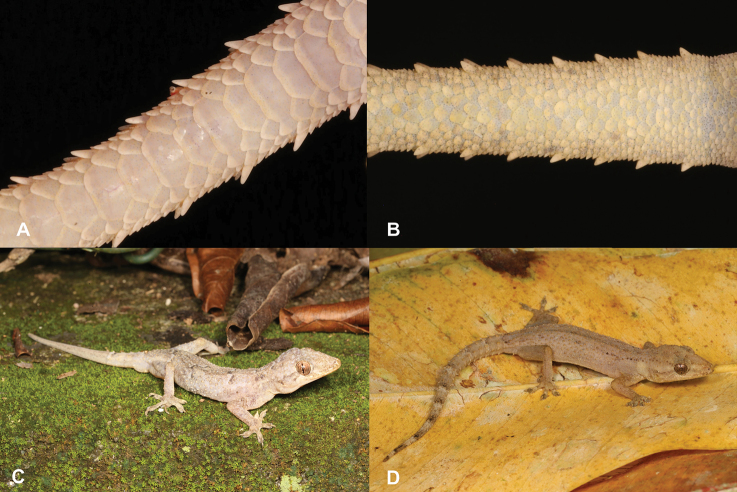
**A** median subcaudals broadened transversely **B** median subcaudals small **C***Hemidactylusmabouia***D***Hemidactyluslongicephalus*. Photographs by Luis M. P. Ceríaco.

**Figure 27.**
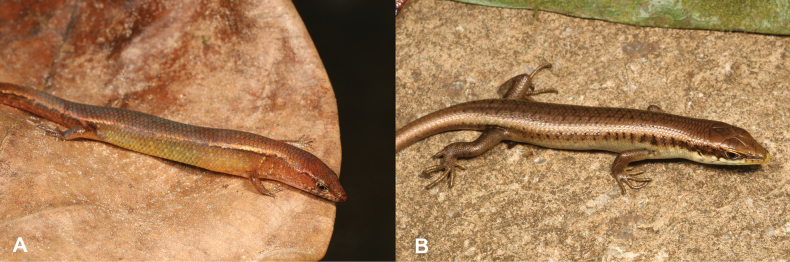
**A** dorsal scales smooth and small limbs and digits **B** dorsal scales keeled and well-developed limbs and digits. Photographs by Luis M. P. Ceríaco.

**Figure 28.**
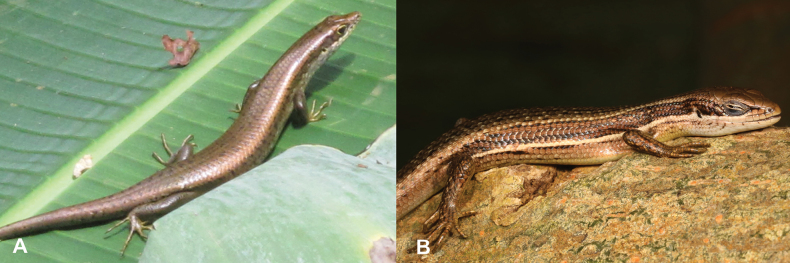
**A***Trachylepisadamastor***B***Trachylepisaffinis*. Photographs by Luis M. P. Ceríaco.

**Figure 29.**
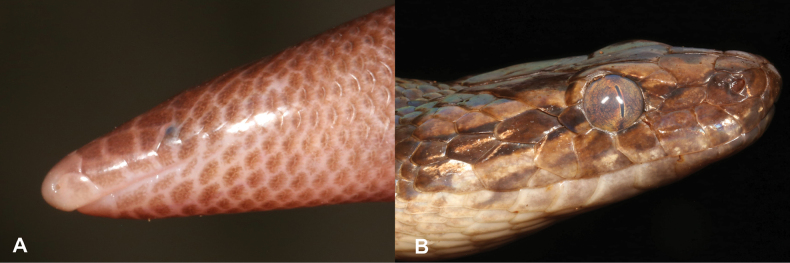
**A** eyes rudimentary to non-visible **B** eyes well developed and visible. Photographs by Luis M. P. Ceríaco.

**Figure 30.**
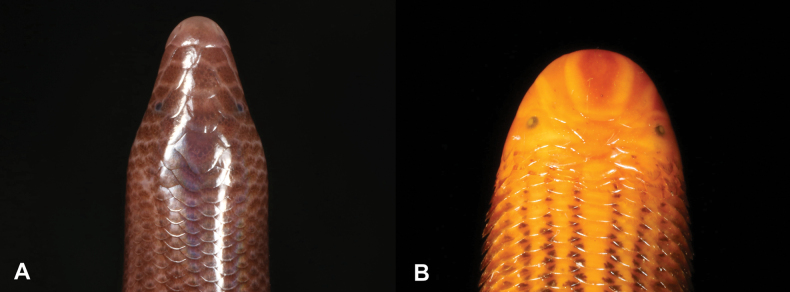
**A** acuminate snout and rostral scale roundish **B** short head and rostral scale in the shape of a fingernail. Photographs by Luis M. P. Ceríaco.

**Figure 31.**
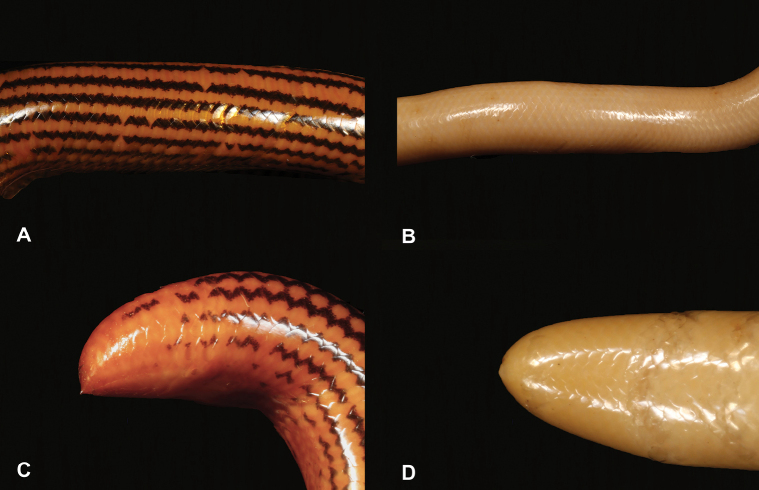
**A** yellow coloration with black stripes **B** pink to beige coloration without stripes **C** presence of a spike at the posterior end of the tail **D** absence of a spike at the posterior end of the tail. Photographs by Luis M. P. Ceríaco.

**Figure 32.**
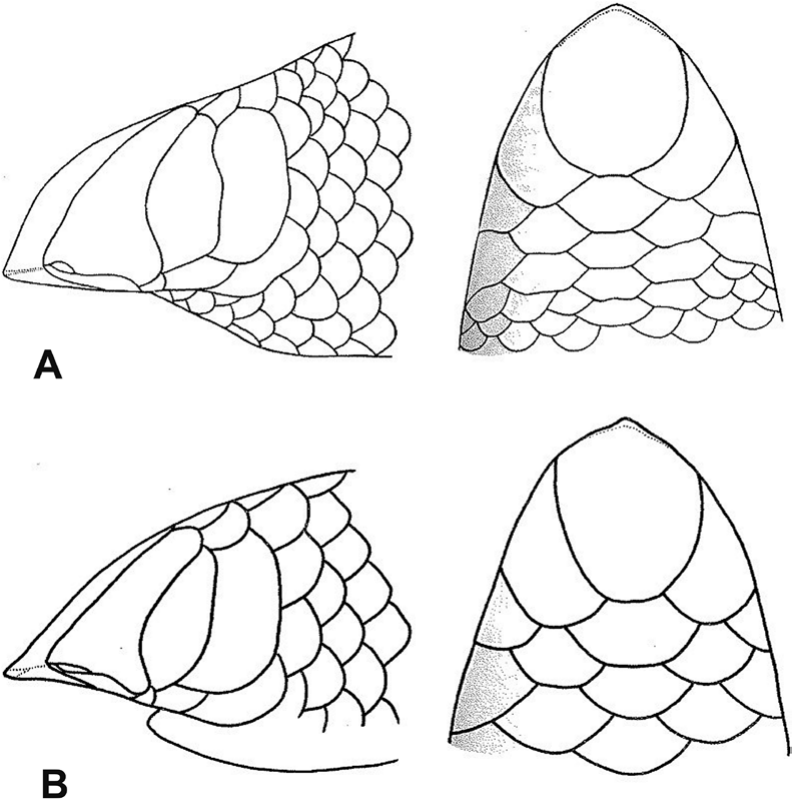
**A***Letheobianewtoni***B***Letheobiafeae* (adapted from Roux-Estève 1974). Photographs by Luis M. P. Ceríaco.

**Figure 33.**
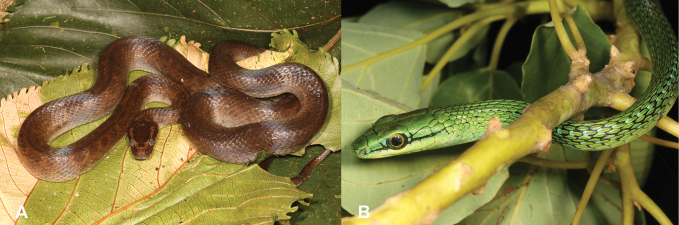
**A***Boaedonmendesi***B***Hapsidophrysprincipis*. Photographs by Luis M. P. Ceríaco.

### ﻿DNA barcoding library

We obtained the full barcode sequence (COI, 658 bp) for 79 specimens, including 50 reptiles of 21 species and 29 amphibians of 9 species (Table 1). Genetic distances between species ranged from 1.3% between *Hyperoliusdrewesi* and *Hyperoliusmolleri* to 26.8% between *Schistometopumthomense* and *Ptychadenanewtoni* in amphibians; and from 6.5% between *Letheobianewtoni* and *Letheobiafeae* to 27.9% between *Panaspisthomensis* and *Philothamnusthomensis* in reptiles. Analysis with the BOLDBIN system yielded nine BINs for amphibians and 23 BINs for reptiles, congruent with the morphological identifications. Only two species presented with two BINs each: *Hemidactyluslongicephalus* and *Letheobianewtoni*. Of the 32 generated BINs, 25 BINs of 22 species are unique to this dataset, with only *Schistometopumthomense*, *Pelusioscastaneus*, *Ptychadenanewtoni*, *Leptopelispalmatus*, *Phrynobatrachusdispar*, *Hyperoliusdrewesi*, and *Hemidactylusmabouia* having COI DNA barcodes of other specimens previously sequenced and BINs attributed. Our results provide the first DNA barcodes for 19 reptiles and 4 amphibian species.

## ﻿Discussion

The illustrated keys provided here aim to facilitate a rapid, accurate, and easy identification of the amphibians and reptiles occurring in São Tomé and Príncipe, serving as a baseline for future ecological studies and surveys, as well as conservation actions. Moreover, they will serve as an important support for the work of the forthcoming generations of researchers studying the biodiversity of these islands. In most cases, morphological identification is sufficient to answer the need of researchers, conservationists, and local authorities and constitutes a rapid and inexpensive method. The species occurring in each island are taxonomically diverse, belonging to different families and genera, and even the taxa that belong to the same genus (maybe with the exception of the members of the genus *Letheobia* in both islands and *Schistometopum* in São Tomé Island) present several conspicuous morphological characters that allow a rapid identification by even a non-herpetologist in most of the situations.

Notwithstanding, DNA barcodes may play an important role in the identification of juveniles lacking good diagnosable traits, amphibian eggs, and larval individuals, and of species with very cryptic morphological variation (e.g., members of the genus *Letheobia* in both islands and *Schistometopum* in São Tomé Island). Moreover, barcodes can be useful to identify poorly preserved and/or partly digested specimens originated from stomach contents or scats of other animals, or to identify animals’ parts and/or animal products being trafficked. However, attention is always needed, and results may sometimes require additional evidence to confidently link a given BIN to a taxon. Single gene methods for species delimitation, such as the use of a single mitochondrial gene as COI, presents some caveats that need to be considered (Dufresne and Jablonski 2022). While BOLD BINs approaches are originally designed for specimen identification, not species delimitation, some abuses and misinterpretations have occurred, leading users to consider BINs as surrogates for taxa (Meier et al. 2021). In our results, most of the BINs were in accordance with the previous taxonomic identification of the respective specimen, but they disagreed in two cases, *Hemidactyluslongicephalus* and *Letheobianewtoni*, in which the BOLDBIN system provided two different BINs for each taxon. This is mostly explained by the existence of intraspecific diversity within the São Tomé population of these species, which can be a result of some degree of geographic isolation between the sequenced specimens. Subsequent morphological analysis of the barcoded specimens of these two taxa, as well as sequencing of additional mitochondrial and nuclear genes which were run against existing phylogenies of the respective groups (Hedges et al. 2014; Ceríaco et al. 2020b) confirmed that the barcoded specimens represent only two taxa, *H.longicephalus* and *L.newtoni*, and no cryptic diversity exists within each taxon. Also, the use of a single mitochondrial gene makes an unambiguous identification impossible in the case of hybrid populations, such as those reported for the species of *Hyperolius* and *Schistometopum* on São Tomé Island (Bell et al. 2015; Bell and Irian 2019; O’Connell et al. 2021). For such cases, nuclear markers are needed to confidently assess their identification. When considering the previously existing DNA sequences, we also found that the specimen identified as *Schistometopumthomense* in Zhang and Wake (2009) groups with our single specimen of *Schistometopumephele* sharing the BINBOLD:AAN0016, both showing a divergence above 3% from all the five specimens of *Schistometopumthomense* in our dataset. This inconsistency roots in an understandable misidentification by Zhang and Wake (2009), as *S.ephele* was at that time still considered as a synonym of *S.thomense* (see O’Connell et al. 2021).

When a solid, complete, and taxonomically well-curated DNA barcode reference library exists, DNA metabarcoding analyses will allow a more detailed and complete glimpse to the understanding of prey patterns in both native and invasive predators (Pompanon et al. 2012; Forin-Wiart et al. 2018; Sousa et al. 2019; Mata et al. 2021; Silva et al. 2021). This is critical for our knowledge and conservation of São Tomé and Príncipe herpetofauna, as the ecological role of the amphibians and reptiles in the local food chain is mostly unknown. This is currently a major information gap because some species may be negatively affected by invasive predators (Bell et al. 2022a; Ceríaco et al. 2022), while others may be feeding on the invasive mammal populations (Ceríaco et al. 2017). DNA barcoding is a relevant method in forensics and to monitor illegal trafficking and has been successfully applied in many regions of the world, for both fauna and flora (Li et al. 2017; Smart et al. 2021; Gostel and Kress 2022). This approach can be of special relevance for the case of the Endangered São Tomé Cobra-Preta, *Najaperoescobari*, for which reports indicate that certain body parts (fat and meat) are being nationally commercialized and internationally trafficked for their assumed benefits for traditional medicines (Ceríaco et al. 2017, 2022).

More recently, environmental DNA approaches have been employed to contribute to the survey of vertebrate species, including amphibians and reptiles (Ficetola et al. 2019; Buxton et al. 2022; Moss et al. 2022; Nordstrom et al. 2022). These approaches can, theoretically, be faster and less dependent on taxonomic expertise (Ruppert et al. 2019) and have been used to try to document the presence of rare and ecologically cryptic and difficult to observe taxa (Rojahn et al. 2021), as well as invasive taxa (Mahon and Jerde 2016). The effectiveness of environmental DNA approaches to survey amphibians and reptiles in the wild is not generalized across the different taxonomic and functional groups, being much more effective for the case of strictly aquatic amphibians (Buxton et al. 2022; Moss et al. 2022) but tends to be less complete for the case of more terrestrial amphibians and reptiles (Kyle et al. 2022; Nordstrom et al. 2022; pers. obs.). Despite its current caveats, environmental DNA is being perceived as an important tool for future studies on African biodiversity (Heyden 2022), and could act as an important component for surveys in usually logistically difficult areas in the islands of São Tomé and Príncipe, where the traditional surveys are highly impacted by the terrain and climatic harsh conditions.

While DNA barcoding is a powerful and useful tool to answer multiple ecological questions, the traditional taxonomic practice remains the fundamental part of biological research and it is impossible to be substituted by any novel technical approaches (Engel et al. 2022). This work is itself a proof of this, as the assembling of this solid and trustworthy DNA barcoding library was entirely dependent on historical and modern taxonomic works that extensively reviewed the identity of all occurring herpetological taxa (Bell et al. 2022a; Ceríaco et al. 2022) and the collection of specimens subsequently deposited in public accessible natural history collections (Rocha et al. 2014). Being one of the taxonomically most well-known and intensively reviewed herpetofauna of Africa, without many taxonomic uncertainties, a considerable number of available specimens and tissue samples and a relatively modest number of occurring taxa, the herpetofauna of São Tomé and Príncipe presents an ideal case for assembling a complete and trustworthy country-wide DNA barcoding library. For the São Tomé and Príncipe birds, a similar group in terms of stable taxonomy, available specimens and tissue samples and a relatively manageable number of taxa (Melo et al. 2022) are another perfect candidate group for the creation of such a library. For other vertebrate groups such as mammals (Rainho et al. 2022) and fishes (da Costa et al. 2022), the rapid assembly of a complete and trustworthy country-wide DNA barcoding library is made difficult by the considerable number of taxonomic uncertainties plaguing some of its species complexes, too large numbers of occurring taxa, and few readily available samples. This situation is even more striking for the case of invertebrates for which the available taxonomic data and for which more fieldwork, collection of new vouchers, and detailed taxonomic revisions are urgently needed (Bell et al. 2022b; Crews and Esposito 2022; Dijkstra et al. 2022; Mendes and Bivar-de-Sousa 2022; Nève et al. 2022; Panisi et al. 2022). As noted by Edward O. Wilson (2017) and Engel et al. (2022), “more [taxonomists’] boots on the ground” are needed to overcome this current taxonomic impediment and provide the basis for the preservation of the unique biodiversity of these islands.

## ﻿Additional information

### Author ORCIDs

Luis Miguel Pires Ceríaco https://orcid.org/0000-0002-0591-9978

Mariana Pimentel Marques https://orcid.org/0000-0002-1712-2632

Ana Carolina Andrade de Sousa https://orcid.org/0000-0003-3435-8775

Joana Veríssimo https://orcid.org/0000-0002-8721-9376

Pedro Beja https://orcid.org/0000-0001-8164-0760

Sónia Ferreira https://orcid.org/0000-0002-6884-3966

### Data availability

All of the data that support the findings of this study are available in the main text.
